# Sepsis Biomarkers: Advancements and Clinical Applications—A Narrative Review

**DOI:** 10.3390/ijms25169010

**Published:** 2024-08-19

**Authors:** Rong-Rong He, Guo-Li Yue, Mei-Ling Dong, Jia-Qi Wang, Chen Cheng

**Affiliations:** 1 Graduate School, Tianjin University of Traditional Chinese Medicine, Tianjin 301617, China; joint-herongrong@simm.ac.cn (R.-R.H.); yueguoli@simm.ac.cn (G.-L.Y.); 2 School of Chinese Materia Medica, Guangdong Pharmaceutical University, Guangzhou 510006, China; dongmeiling@simm.ac.cn; 3 State Key Laboratory of Drug Research, Shanghai Institute of Materia Medica, Chinese Academy of Sciences, Shanghai 201203, China; wangjiaqi1@simm.ac.cn

**Keywords:** sepsis, biomarkers, diagnosis, prognosis, clinical applications

## Abstract

Sepsis is now defined as a life-threatening syndrome of organ dysfunction triggered by a dysregulated host response to infection, posing significant challenges in critical care. The main objective of this review is to evaluate the potential of emerging biomarkers for early diagnosis and accurate prognosis in sepsis management, which are pivotal for enhancing patient outcomes. Despite advances in supportive care, traditional biomarkers like C-reactive protein and procalcitonin have limitations, and recent studies have identified novel biomarkers with increased sensitivity and specificity, including circular RNAs, HOXA distal transcript antisense RNA, microRNA-486-5p, protein C, triiodothyronine, and prokineticin 2. These emerging biomarkers hold promising potential for the early detection and prognostication of sepsis. They play a crucial role not only in diagnosis but also in guiding antibiotic therapy and evaluating treatment effectiveness. The introduction of point-of-care testing technologies has brought about a paradigm shift in biomarker application, enabling swift and real-time patient evaluation. Despite these advancements, challenges persist, notably concerning biomarker variability and the lack of standardized thresholds. This review summarizes the latest advancements in sepsis biomarker research, spotlighting the progress and clinical implications. It emphasizes the significance of multi-biomarker strategies and the feasibility of personalized medicine in sepsis management. Further verification of biomarkers on a large scale and their integration into clinical practice are advocated to maximize their efficacy in future sepsis treatment.

## 1. Introduction

Sepsis is life-threatening organ dysfunction caused by a dysregulated host response to microbial infection [1]. This critical illness imposes a substantial burden, evidenced by its high incidence and mortality rates. In 2017, over 48.9 million individuals globally were diagnosed with sepsis, and approximately one-fifth of all deaths were attributed to this condition [2]. Fleischmann-Struzek et al. conducted a meta-analysis revealing that hospital-acquired sepsis occurred in 189 cases per 100,000 individuals annually, with a mortality rate of 26.7% [3]. Owing to its significant impact, the World Health Organization has identified sepsis as a health priority of utmost importance.

Sepsis 1.0, the initial definition of sepsis, was proposed by the Society of Critical Care Medicine (SCCM) and the American College of Chest Physicians (ACCP) in 1991. Acute infections that meet two or more systemic inflammatory response syndrome (SIRS) criteria are defined as SIRS. In addition, severe sepsis and septic shock criteria have been established based on the severity of the condition [3,4]. The introduction of Sepsis 2.0 in 2001 did not significantly change the definition; however, it incorporated complex clinical diagnostic criteria that were deemed to have limited clinical utility, thus resulting in the ongoing utilization of the Sepsis 1.0 criteria. While Sepsis 1.0 standardized the diagnostic criteria for sepsis, subsequent applications indicated that SIRS is not a compulsory condition for sepsis diagnosis, underscoring the necessity for new diagnostic criteria [5].

Sepsis 3.0 was introduced in 2016 by a panel convened by the European Society of Intensive Care Medicine (ESICM) and the SCCM following extensive deliberations [1,6,7]. Sepsis 3.0 is typically diagnosed by the Sequential Organ Failure Assessment (SOFA) score. However, in clinical settings, a quick SOFA score is recommended in the initial evaluation of patients who have been infected or are suspected of being infected. If the quick SOFA score is equal to or higher than two points, a more detailed assessment of the patient’s status is carried out using the SOFA score. In the presence of an infection, a patient’s SOFA score rises by two points above their baseline, indicating organ dysfunction, thus indicating sepsis. Moreover, the 2016 agreement abolished severe sepsis. Instead, it redefined septic shock as requiring the administration of vasopressors to maintain a mean arterial pressure of at least 65 mmHg after receiving adequate fluid replenishment. This should be coupled with a blood lactate level exceeding 2 mmol/L [8,9]. Currently, sepsis treatment primarily includes antimicrobial therapy and general supportive care [10]. Early treatment following the diagnosis of sepsis can improve the success rate of interventions [11]. Many therapeutic agents for sepsis fail, potentially due to the erroneous assumption that a single therapeutic strategy can equally counteract all clinical presentations, comorbidities, and diverse prognostic capabilities in a heterogeneous patient population.

The existing diagnostic criteria evaluate organ damage associated with sepsis but lack markers directly indicative of infection. Although pathogen detection is widely accepted as the most reliable method for diagnosing infections, clinical microbiological culture and identification processes are typically slow, requiring 2−3 days or longer, and often produce low positivity rates. The absence of early specific diagnostic markers results in roughly a quarter of sepsis cases being diagnosed belatedly, resulting in suboptimal treatment outcomes and poorer prognosis. This underscores the critical need for the development of early sepsis diagnostic markers [12,13]. There is also a demand for biological markers capable of monitoring the body’s metabolic status and continuously assessing patient severity, particularly in cases related to infection [14].

Over 250 sepsis biomarkers have been identified in recent years, with ongoing discoveries. Sepsis biomarkers have garnered significant attention in recent years due to their potential for improving the early diagnosis and prognosis of sepsis and septic shock, especially in ICU settings where these conditions are prevalent. Recent studies published from 2020 to 2023 have highlighted novel biomarkers and their clinical applications, underscoring the evolving landscape of sepsis biomarker research (Table 1). This review offers a comprehensive overview of key biomarkers utilized in recent years, elucidating their clinical implications and introducing emerging biomarkers with promising research outcomes. It emphasizes the advancement of multi-biomarker strategies and the potential for personalized medicine in sepsis management. Furthermore, it explores the necessity for the large-scale validation of biomarkers and their eventual integration into clinical practice, with the aim of enhancing treatment outcomes.

## 2. What Is an Ideal Sepsis Biomarker?

An ideal sepsis biomarker should have high sensitivity and specificity to detect sepsis early for accurate diagnosis [21]. It should provide rapid and precise results through accessible testing methods, aiding healthcare providers in timely interventions [16]. This biomarker would serve as both a diagnostic tool and a monitoring parameter for the real-time assessment of sepsis progression and treatment effectiveness [22]. Additionally, it would offer valuable prognostic information for patient outcomes, ensuring reliable performance across diverse populations and clinical settings. Cost-effective and non-invasive, the ideal biomarker eases financial burdens on healthcare systems and improves resource allocation for sepsis management. Thorough validation and clear guidelines are crucial for maximizing the utility of these biomarkers in effective sepsis management [23,24].

## 3. Methods

### 3.1. Inclusion and Exclusion Criteria

The inclusion criteria for this review encompassed studies investigating biomarkers associated with sepsis diagnosis and prognosis, specifically focusing on clinical trials conducted on human subjects evaluating the effectiveness and reliability of biomarkers in diagnosing or prognosticating sepsis. These studies were required to be published in peer-reviewed journals and provide adequate data on the study design, participant characteristics, and outcomes. Exclusion criteria involved studies that did not emphasize sepsis biomarkers, non-peer-reviewed articles, and studies with incomplete or ambiguous data.

### 3.2. Search Strategies

A thorough literature search was conducted in the databases PubMed and Web of Science. The search strategy utilized a combination of keywords and MeSH terms, including “sepsis”, “biomarkers”, “diagnosis”, “prognosis”, and “clinical”. Boolean operators (AND, OR) were employed to enhance the search, concentrating on studies published up to the year 2024, with particular focus on clinical research articles from 2021 to 2024. The complete search strategy for each database is outlined below.

PubMed: (“sepsis”) AND (“biomarkers” OR “diagnostic markers”) AND (“prognosis” OR “outcomes”) AND (“clinical”).

Web of Science: (“sepsis”) AND (“biomarkers” OR “diagnostic markers”) AND (“prognosis” OR “outcomes”) AND (“clinical”).

### 3.3. Study Selection

Two independent reviewers screened the titles and abstracts of the retrieved studies to identify those that met the inclusion criteria. Subsequently, the full-text articles of potentially eligible studies underwent assessment for final inclusion. Any discrepancies between the reviewers were resolved through discussion or consultation with a third party.

### 3.4. Data Collection

Data were extracted from the included studies using a standardized data extraction form. The extracted data included study characteristics (e.g., author, year, design), participant details (e.g., sample size, demographics), studied biomarkers, and key findings concerning the diagnosis and prognosis of sepsis.

## 4. Biomarkers for Sepsis Diagnosis

In this review, more than 100 studies were referenced to provide a comprehensive overview of sepsis diagnosis biomarkers. These studies were selected based on their relevance to the core themes discussed, focusing on the latest advancements in sepsis biomarker research and highlighting the progress and clinical implications.

### 4.1. Commonly Used Diagnostic Biomarkers

#### 4.1.1. C-Reactive Protein (CRP)

CRP is a five-part protein synthesized by the liver when exposed to inflammatory cytokines like IL-6 and IL-1β. This protein triggers the complement system and encourages platelets, monocytes, and endothelial cells to become active [25]. In healthy individuals, CRP levels are typically low, but can increase within 4–6 h and rise significantly within 24–48 h in response to inflammation or acute infection [22].

In a recent study, a group of researchers analyzed data from a cohort of 279 ICU patients to evaluate 53 biomarkers and their ability to differentiate between sepsis and non-septic SIRS. The results showed that none of the biomarkers or combinations of biomarkers performed better than CRP, which had the area under the receiver operating characteristic (AUROC) of 0.76 (95% CI [0.68–0.84]) [26]. The AUROC, also known as the AUC (area under the curve), indicates the ability of a biomarker to distinguish between positive and negative cases; a value closer to one suggests better performance. Another study indicated that CRP levels above 59.25 mg/L (sensitivity 74.4% and specificity 65.4%) could diagnose Gram-negative bacterial sepsis, while lower values suggested Gram-positive bacterial sepsis [27]. Septic patients showed increased levels of CRP (septic patients: 182.9 ± 132.9 mg/mL; non-septic patients: 93.46 ± 117.6 mg/mL; *p* = 0.030) and sepsis index (septic patients: 0.19 ± 0.19; non-septic patients: 0.08 ± 0.08; *p* = 0.010) on day +3 after admission [28]. Another study suggested a relationship between neonatal sepsis and maternal third-trimester CRP, and indicated the possibility that this effect might be dependent on the gestational age [29].

CRP serves as a valuable biomarker in the prompt identification of sepsis, boasting a high degree of sensitivity, yet indicating restricted specificity [15]. Guidelines from organizations like the Surviving Sepsis Campaign (SSC) emphasize CRP as a crucial biomarker in the diagnosis and management of sepsis [1]. It is frequently used in postoperative settings to monitor recovery, with levels typically remaining elevated post-surgery before gradually declining. However, its diagnostic accuracy can vary significantly depending on the clinical setting and patient population. In cases of trauma, surgery, and autoimmune diseases, elevated CRP levels do not necessarily suggest the presence of sepsis, as the elevation is often associated with sterile inflammation. Conversely, CRP testing may produce false-negative results in localized infections, where the inflammatory reaction may not be pronounced enough to trigger a substantial rise in CRP levels [30]. Although CRP on its own possesses moderate diagnostic accuracy for differentiating between sepsis and non-sepsis, its efficacy is greatly improved when combined with PCT [31]. While no single sepsis biomarker may be completely ideal, the discovery and study of multiple biomarkers can at least help identify sepsis patients who require closer monitoring, facilitating timely diagnosis and treatment [32].

Although elevated levels of CRP in the blood can indicate inflammation in the body, it is not specific to differentiating between bacterial and viral infections [33]. CRP levels can be elevated in both bacterial and viral infections, as well as other inflammatory conditions, making it a nonspecific marker of inflammation. While CRP levels may be higher in bacterial infections compared to viral infections in some cases, it is not reliable enough to definitively distinguish between the two. In clinical practice, healthcare providers typically use a combination of tests, including CRP levels, white blood cell counts, and specific pathogen tests, to help differentiate between bacterial and viral infections. A 2022 study analyzed the effectiveness of using the estimated C-reactive protein velocity (eCRPv) as a novel marker to differentiate between acute bacterial and viral infections. The study found that eCRPv values were significantly higher in patients with bacterial infections compared to those with viral infections, particularly in cases with intermediate CRP levels, where the diagnosis is often uncertain. This finding suggests that an eCRPv > 4 mg/L/h is a strong indicator of bacterial infection, which could expedite the appropriate therapeutic management in acute febrile illnesses [34].

#### 4.1.2. Procalcitonin (PCT)

Upon detection of bacteria, particularly Gram-negative bacteria, the body activates immune cells like macrophages and monocytes, leading to the release of cytokines such as IL-1, IL-6, and TNF-α. These cytokines stimulate the production of PCT in the liver and other tissues. PCT is initially synthesized as an inactive precursor protein known as pre-procalcitonin [35]. This precursor undergoes proteolytic cleavage in response to bacterial toxins and inflammatory mediators, leading to the release of the active form of PCT. In viral or mild bacterial infections, PCT levels remain stable or slightly elevated. Conversely, during severe infections or sepsis, PCT levels begin to rise within 2–4 h, peaking at 24 h with an increase of hundreds or even thousands of times [17]. The measurement of urine PCT levels can be informative in septic patients, especially in cases of urinary tract infections or sepsis of urinary origin. Additionally, PCT levels in cerebrospinal fluid (CSF) can aid in identifying central nervous system infections like bacterial meningitis in septic patients.

A meta-analysis from 2011 to 2022 included 10 out of 2457 studies to evaluate the clinical value of PCT [36]. It was shown that PCT levels were significantly elevated in sepsis patients (29.3 ± 85.3 ng/mL) compared to the control groups (0.34 ± 8.6 ng/mL) [37]. Recent research indicates that PCT has a significantly higher AUC value compared to standard infection indicators, underscoring its high diagnostic value for sepsis [38,39]. Data from six studies showed that, despite significant heterogeneity, the sensitivity and specificity of PCT for diagnosing sepsis in adults were 0.73 (95% CI [0.59, 0.87]) and 0.77 (95% CI [0.66, 0.88]), respectively. These values indicate that the diagnostic performance of PCT is still quite good. According to five studies, the positive and negative predictive values of PCT for diagnosing sepsis were 1.26 (95% CI [0.72, 1.79]) and 0.51 (95% CI [0.34, 0.68]), respectively [37,40,41,42,43]. PCT demonstrated superior diagnostic accuracy for predicting positive blood cultures, with an AUC of 0.72 and a diagnostic odds ratio of 3.64, outperforming both lactate and high-sensitivity CRP. Additionally, PCT levels above 2.44 ng/mL (sensitivity 77.1% and specificity 68.4%) can indicate Gram-negative bacterial sepsis, while lower values suggest Gram-positive bacterial sepsis [27]. Higher PCT levels markedly increased the likelihood of positive Gram-negative bacteremia, with a diagnostic odds ratio of 6.44, and showed even better performance in an expanded cohort [41]. A total of 295 patients with sepsis admitted to the hospital from January 2021 to December 2022 were collected and divided into a survival group and a death group according to their 28-day survival status. The 28-day survival rate of the low-PCT-level group was 93.4% and that of the high-PCT-level group was 51.7% at the node of 2.85 ng/mL, and the difference between the two groups was statistically significant (χ^2^ = 63.437, *p* < 0.001). The study shows that the PCT levels of the non-survivors were higher than those of the survivors (6.25 ± 1.91 vs. 4.03 ± 1.26, t = −11.546, *p* < 0.001) [44].

Additionally, PCT can be utilized to inform antibiotic therapy. Clinical guidelines recommend discontinuing antibiotics when PCT levels fall below 0.5 ng/mL or decrease by 80% from their peak levels. If PCT levels remain elevated (exceeding 0.5 ng/mL), it is recommended to persist with or modify the antibiotic regimen until the levels decline. These standardized procedures have demonstrated efficacy in reducing the overall utilization of antibiotics and enhancing antibiotic stewardship [45]. Using PCT as a biomarker can lead to more targeted antibiotic therapy, helping to reduce unnecessary antibiotic use, minimize the development of antibiotic resistance, and improve patient outcomes. By closely monitoring PCT levels, healthcare providers can make informed decisions regarding the continuation or discontinuation of antibiotics based on the likelihood of a bacterial infection. For instance, the SSC guidelines suggest using PCT levels to help make decisions regarding the initiation and duration of antibiotic therapy in patients with sepsis and septic shock. These guidelines emphasize the importance of a multifaceted approach to sepsis management, of which PCT testing is a key component. Further investigation is imperative to validate the applicability of these strategies in broader clinical settings.

#### 4.1.3. Interleukin-6 (IL-6)

IL-6, discovered in 1986, is a cytokine produced by various cells, including immune cells such as monocytes and macrophages, as well as non-immune cells like fibroblasts and endothelial cells [18]. Normally, serum concentrations of IL-6 range from 1 to 25 pg/mL, but in sepsis, they can exceed 1 ng/mL. As an early inflammatory mediator, IL-6 facilitates the proliferation and differentiation of T and B lymphocytes and stimulates the synthesis and secretion of acute-phase proteins, peaking within 2 h of an inflammatory response. IL-6 exhibits a quicker response to infections than CRP and PCT, establishing its reputation as a prominent early biomarker for sepsis, notably in China. Despite not being universally incorporated in sepsis management protocols, several studies have highlighted its prospective value as a sepsis biomarker. Recent research suggests that IL-6 can independently predict the diagnosis of sepsis, with a sensitivity of 68%, a specificity of 83%, and an AUC of 0.764 [46]. A study evaluated the clinical utility of IL-6, PTX3, and PCT in patients with sepsis and septic shock by measuring the serum concentration of these markers in 142 subjects. The study determined the optimal cutoff values for sepsis and septic shock, demonstrating that IL-6 levels could effectively distinguish between the two conditions [47]. One study investigated the relationship between T lymphocyte subsets, IL-6, and PCT, and the severity of sepsis in 120 patients at Baoding No. 1 Central Hospital. Results showed that, as the sepsis severity increased, CD3+, CD4+, and CD4+/CD8+ levels decreased, while IL-6 and PCT levels rose significantly. The study concluded that changes in these biomarkers correlate strongly with sepsis severity and can predict recovery outcomes, underscoring their clinical significance [48]. Research is ongoing to further evaluate the clinical significance of IL-6 and its incorporation into sepsis management guidelines. As more evidence accumulates, we may see increased adoption of IL-6 testing in the clinical setting to improve sepsis management and patient outcomes.

#### 4.1.4. High-Mobility Group Box 1 (HMGB1)

HMGB1 is a non-histone nuclear protein that plays a crucial role in inflammation. It can be secreted by activated macrophages or released during cell necrosis and apoptosis. Identified in 1999 as a late-stage mediator of sepsis, HMGB1 acts as a damage-associated molecular pattern (DAMP), prolonging inflammation by activating macrophages via Toll-like receptor 4 (TLR4) and the receptor for Advanced Glycation Endproduct (RAGE) pathways [49,50,51,52]. Elevated levels of HMGB1 in the later stages of sepsis are often associated with unfavorable outcomes, especially in patients with concurrent chronic inflammatory conditions, leading to higher mortality rates [53]. HMGB1 shows promise as a biomarker for sepsis diagnosis and prognosis assessment. Additionally, it has the potential to predict the duration of ICU stays for patients with septic shock.

Although HMGB1 is not yet included in standard sepsis guidelines, research on its utility as a biomarker is ongoing. Recent studies have shown that, once HMGB1 levels reach a critical threshold of 1.20 ng/mL, and an AUC of 0.58 (0.35–0.78) [54]. At a critical concentration of 15.27 ng/mL, HMGB1 demonstrates a 100% sensitivity and an 83.33% specificity in predicting the prolonged ICU stay of sepsis patients for more than five days, achieving an AUC of 0.958 [55]. The clinical data of 209 patients with sepsis were analyzed retrospectively, which showed that the levels of SII, IL-35, and HMGB-1 were significantly positively correlated with the severity of sepsis (*p* < 0.05), and significantly positively correlated with the prognosis of patients with sepsis (*p* < 0.05) [56]. Some studies have proposed incorporating HMGB1 testing into a multimodal approach to sepsis diagnosis and management, but further validation and consensus are needed before widespread implementation.

#### 4.1.5. Pancreatic Stone Protein (PSP)

PSP is an acute-phase reactant secreted by pancreatic acinar cells. Fluctuations in PSP levels are closely linked to the progression of sepsis, serving as a basis for early diagnosis and timely treatment [57,58]. Meta-analyses and studies have demonstrated that the pooled sensitivity and specificity of PSP for diagnosing sepsis range from 0.77 to 0.86 and 0.73 to 0.78, respectively, comparable to PCT and CRP [59]. A meta-analysis from 1966 to 2019, which encompassed 5 out of 17 studies, aimed to evaluate the clinical significance of PSP. It has been shown that PSP levels in sepsis patients (44.18 ng/mL) are significantly higher than those in healthy subjects (10.4 ng/mL), suggesting that PSP is a promising biomarker for the early diagnosis of infections in hospitalized patients [60]. Studies indicate that analyzing serum PSP levels within 14 days post-injury reveals a threefold increase within the first 72 h before clinical sepsis diagnosis and a more than tenfold increase within the first 48 h before septic shock diagnosis compared to baseline levels [61]. A multicenter prospective observational clinical study conducted in 14 ICUs across France, Switzerland, Italy, and the UK found that, in 243 patients, PSP levels began to rise five days before the clinical diagnosis of sepsis. The AUC for PSP (0.75) was similar to that for CRP and PCT, suggesting that continuous PSP measurement could have potential clinical benefits in treating critically ill patients with hospital-acquired sepsis [62]. A prospective multicenter cohort study showed that PSP was a predictor of the severity of infection and in-hospital death in a specific subset of ICU patients with complicated abdominal surgery or acute necrotizing pancreatitis [63]. Point-of-care testing for PSP is quick and convenient, showing the strong potential for early sepsis identification, although further validation through large-scale studies is imperative. While PSP shows promise as a biomarker for sepsis, it is important to note that its clinical use is not yet widespread. Further research is needed to establish the utility of PSP in routine clinical practice and to determine its sensitivity and specificity compared to existing biomarkers for sepsis. As for guidelines or recommendations regarding the use of PSP as a sepsis biomarker, there are currently no specific guidelines or recommendations from major medical organizations. However, ongoing research and clinical trials may provide more insights into the potential role of PSP in the management of sepsis in the future.

#### 4.1.6. Presepsin

Presepsin is a novel immune biomarker that exists as a soluble form of CD14. CD14 is a surface glycoprotein, part of the Toll-like receptor (TLR) family, expressed on macrophages and monocytes, with a high affinity for bacterial ligands such as lipopolysaccharides [64]. While presepsin is not yet in widespread use, it has been assessed for its utility in diagnosing bacterial sepsis, guiding antibiotic treatment decisions, and evaluating sepsis prognosis. Research has revealed that presepsin levels exceeding 946 ng/L are strongly associated with Gram-negative bacterial sepsis. Conversely, a threshold of 600 ng/L was found to be ineffective in differentiating between Gram-positive and Gram-negative infections. These findings suggest that higher presepsin levels are more indicative of Gram-negative bacterial infections [65]. However, the range of values and their predictive accuracy vary across different studies [65]. A multicenter prospective cohort trial investigated whether a presepsin-guided strategy could safely shorten antibiotic treatment in patients with septic shock. In the study, 656 patients were divided into a presepsin group, where antibiotics were discontinued based on presepsin levels, and a control group that followed standard guidelines. The study found that the presepsin group had significantly more antibiotic-free days and reduced hospital stay and costs (*p* < 0.001), with no difference in mortality, recurrent infections, or organ failure between the groups [64]. Currently, there are no established guidelines or recommendations for a universally accepted presepsin threshold, pending further research. Comparison of presepsin levels in mouse models of CLP, CL, and LPS-induced sepsis showed that serum presepsin levels are specific to bacterial infection [66]. In addition, PSP was found to be significantly elevated in patients presenting with severe COVID-19, and levels above 775 pg/mL were significantly associated with in-hospital mortality (sensitivity 73% and specificity 80%) [67]. Presepsin levels increase in response to bacterial infections, making it a valuable biomarker for the early detection of sepsis. Monitoring presepsin levels can assist in assessing the severity and progression of sepsis, guiding treatment decisions, and predicting patient outcomes. However, the incorporation of presepsin testing into routine clinical practice may vary among different healthcare institutions and regions.

#### 4.1.7. Cluster of Differentiation 64 (CD64)

CD64, a type I receptor for the Fc fragment of immunoglobulin G, is constitutively expressed on the surface of monocytes, macrophages, and dendritic cells, and mediates bacterial phagocytosis. Inactivated neutrophils rarely express CD64; however, in neutrophils stimulated by the plasma of sepsis patients, CD64 expression significantly increases. Extensive literature demonstrates that CD64 serves as a valuable marker for the early diagnosis of sepsis in various clinical settings, including emergency departments and ICUs [68]. A study assessing the diagnostic accuracy of the neutrophil CD64, PCT, and IL-6 for sepsis found that CD64 exhibited a sensitivity of 0.88 (95% CI, 0.81–0.92), a specificity of 0.88 (95% CI, 0.83–0.91), and an AUC of 0.94 (95% CI, 0.91–0.96) [69]. A review of articles spanning from 2006 to 2019 evaluated the clinical utility of CD64, demonstrating a pooled sensitivity and specificity of 0.87 and 0.89, respectively, with a summary AUC of 0.94 [70]. A prospective analysis involving 207 patients with hematological diseases (non-infected group, n = 50; locally infected group, n = 67; sepsis group, n = 90) and 26 healthy volunteers evaluated among patients with hematological diseases. According to the absolute neutrophil count (ANC), patients with hematological diseases without infection were divided into the normal ANC, ANC reduced, and ANC deficiency groups. There was no statistically significant difference in the nCD64 index between these three groups (P = 0.586). However, there was a difference in the nCD64 index among the non-infected (0.74 ± 0.26), locally infected (1.47 ± 1.10), and sepsis (2.62 ± 1.60) groups (*p* < 0.001). The area under the diagnosis curve of the nCD64 index, evaluated as the difference between the sepsis and locally infected group, was 0.777, which was higher than for PCT (0.735) and hs-CRP (0.670). The positive and negative likelihood ratios were also better for the nCD64 index than either for PCT or hs-CRP. This study confirmed that the neutrophil CD64 (nCD64) index can be used for the early diagnosis of sepsis in hematological patients [71]. Another study demonstrated that the nCD64 index combined with CRP was superior to CRP, PCT, the nCD64 index, and the nCD64 index plus PCT in predicting the 28-day mortality in sepsis. A multi-marker approach could improve the predictive accuracy and be beneficial for septic patients [72]. Elevated CD64 levels in sepsis patients can indicate an ongoing infection even before the development of clinical symptoms, facilitating early intervention and treatment. Although CD64 is not yet included in routine clinical guidelines for sepsis management, some studies and meta-analyses have highlighted its potential utility as a biomarker. Some institutions and experts in the field may recommend incorporating CD64 measurement into sepsis management protocols, particularly in the context of early identification and risk stratification.

#### 4.1.8. Soluble Triggering Receptor Expressed on Myeloid Cells-1 (sTREM-1)

sTREM-1 is a glycoprotein expressed on the surface of neutrophils, mature monocytes, and macrophages. Infections caused by bacteria can lead to an increased expression of sTREM-1. As a member of the TREM family, sTREM-1 is a promising biomarker for infections due to its quantifiability in bodily fluids like serum, pleural effusion, sputum, and urine. A meta-analysis involving 19 studies and 2,418 patients evaluated the diagnostic value of sTREM-1 in suspected cases of sepsis. The results suggest that sTREM-1 has a moderate diagnostic accuracy for identifying sepsis in high-risk patients, with a combined sensitivity of 0.82 (95% CI, 0.73–0.89) and a specificity of 0.81 (95% CI, 0.74–0.86). Therefore, further large-scale studies are necessary to comprehensively assess the diagnostic precision of sTREM-1 [73]. Recently, a meta-analysis indicated that circulating sTREM-1 showed a high sensitivity (0.85 (95% confidence interval (CI): 0.76–0.91)) and moderate specificity (0.79 (95% CI: 0.70–0.86)) to differentiating sepsis from SIRS. The study showed a high sensitivity (0.80 (95% CI: 0.66–0.89)) and moderate specificity (0.75 (95% CI: 0.69–0.81)) to predicting the 28-day mortality in sepsis, suggesting that circulating sTREM-1 showed diagnostic and prognostic predictive values in sepsis [74]. sTREM-1 has been studied for its potential role in distinguishing bacterial infections from non-infectious inflammatory conditions and guiding antibiotic therapy in sepsis. At present, sTREM-1 is not yet widely adopted in clinical practice, and further studies are needed to establish its clinical utility and standardize its use in routine sepsis management.

### 4.2. Novel Diagnostic Biomarkers

#### 4.2.1. Circular RNAs (circRNAs)

Circular RNAs, first discovered in viruses in 1970s, have been increasingly identified in various cells with the advancement of bioinformatics techniques [75]. They play significant roles in gene transcription and cellular processes such as proliferation, autophagy, and apoptosis, and are closely linked to sepsis [76]. Recent studies propose circRNAs as valuable biomarkers for sepsis diagnosis due to their involvement in immune responses and the regulation of miRNAs [77,78]. A study investigated the role of exosomal circRNAs in sepsis by extracting exosomes from the serum of 25 sepsis patients treated at the Second Hospital of Jilin University from September 2018 to January 2019, as well as from 22 healthy individuals, using ultracentrifugation. The analysis of the circRNA expression was conducted through a microarray analysis. The results identified 132 significantly differentially expressed circRNAs, among which hsa_circRNA_104484 and hsa_circRNA_104670 were notably increased in sepsis patients. These findings suggest that these two circRNAs could potentially serve as novel diagnostic biomarkers [79]. Circular RNAs (circRNAs) have been manifested to be involved in the development of human diseases, including sepsis-associated acute kidney injury (SA-AKI). A recent study identified that circ_0006944 exacerbated SA-AKI development via the miR-205-5p/UBL4A axis, which might be a potential treatment and diagnosis biomarker for SA-AKI [80]. As of now, the use of circRNAs as sepsis biomarkers is still in the research phase, and there is limited incorporation of circRNA testing into clinical guidelines or recommendations for sepsis management. However, with further validation studies and clinical trials, circRNAs may eventually find their place in sepsis guidelines as reliable biomarkers for the early detection, prognosis assessment, and treatment monitoring of septic patients.

#### 4.2.2. HOXA Distal Transcript Antisense RNA (HOTTIP)

ARDS is characterized by rapid fibroproliferative response and early inflammation, with pulmonary fibrosis being a significant determinant of poor prognosis among sepsis-induced ARDS patients. Long non-coding RNAs (lncRNAs) are RNA molecules exceeding 200 nucleotides that do not encode proteins but can interact with DNA, RNA, and proteins to control gene expression. Dysregulation of lncRNAs has emerged as a significant contributor to the pathogenesis of Acute Respiratory Distress Syndrome (ARDS) in sepsis [81]. Specifically, HOTTIP, an lncRNA located on chromosome 7q15.2, has been identified as an inflammation-associated gene. Increased expression of HOTTIP has been associated with the diagnosis of acute gouty arthritis and the excessive secretion of inflammatory mediators [82]. A study involving 118 sepsis patients and 96 healthy controls identified HOTTIP as a risk factor for ARDS development. The study demonstrated that HOTTIP can effectively differentiate between ARDS and non-ARDS patients, with an AUC value of 0.847. Hence, HOTTIP serves as a valuable diagnostic biomarker for ARDS identification in sepsis patients [83]. Further validation studies are needed to establish the clinical utility of HOTTIP as a sepsis biomarker and its potential incorporation into clinical practice guidelines.

#### 4.2.3. microRNA-486-5p

MicroRNAs (miRNAs) are small non-coding RNAs that regulate gene expression post-transcriptionally and are currently under investigation as potential biomarkers for sepsis. MicroRNAs play a vital role as endogenous non-coding post-transcriptional regulators, exerting negative control over target mRNAs through base pairing. Their abnormal expression not only influences the fundamental biological processes of diseases but also serves as potential diagnostic and prognostic indicators due to their evolutionary conservation and stability [84]. The utility of miRNAs as biomarkers for sepsis is under active investigation [85,86,87]. Among the numerous miRNAs, miRNA-486-5p (miR-486-5p), located on human chromosome 8p11.21, emerges as a promising biomarker for sarcopenia in the elderly [88].

In a study spanning from 2016 to 2019, serum levels of miR-486-5p were evaluated in 108 sepsis patients, 60 pneumonia-infected patients, and 101 healthy subjects. The findings demonstrated higher serum miR-486-5p levels in sepsis patients (*p* < 0.001). Notably, serum miR-486-5p not only discriminated sepsis from healthy subjects (AUC = 0.914) but also significantly differentiated sepsis from pneumonia-infected individuals (AUC = 0.814), underscoring its robust potential as a diagnostic biomarker for sepsis. This investigation emphasizes the promise of miR-486-5p as a diagnostic biomarker for sepsis [89]. Furthermore, A meta-analysis involving 50 studies totaling 5225 sepsis patients and 4008 controls, involving 48 miRNAs, evaluated the miRNA diagnostic value in suspected cases of sepsis. The results suggest that TmiRs had a combined AUC of 0.86, with a pooled sensitivity of 0.76 and a specificity of 0.77, indicating that miRNAs had a moderate diagnostic accuracy as a diagnostic biomarker in discriminating sepsis. In addition, they examined individual miRNAs in the overall miRNA library and discovered that miR-155-5p, miR-21, miR-223-3p, miR-146a, and miR-125a were the ones most often used in recent studies. Among all miRNAs, miR-155-5p had the highest AUC of SROC, with a pooled sensitivity of 0.71 (95% CI, 0.67 to 0.75), a pooled specificity of 0.82 (95% CI, 0.76 to 0.86), and a SROC of 0.85, indicating that miRNAs, specifically miR-155-5p, could be useful biomarkers for detecting sepsis [90]. Although miR-486-5p is not yet included in established clinical guidelines or recommendations for sepsis management, ongoing research continues to validate its utility as a biomarker in this setting. As more evidence accumulates supporting the use of miR-486-5p, it may be integrated into future guidelines to enhance the diagnostic and prognostic capabilities in sepsis care.

The biomarkers linked to sepsis diagnosis, along with the limitations and strengths observed in clinical practice, are outlined in Table 2. 

## 5. Biomarkers for Sepsis Prognosis

### 5.1. Commonly Used Prognostic Biomarkers

#### 5.1.1. Pentraxin-3 (PTX-3)

PTX-3, a biomarker belonging to the pentraxin protein family, plays a crucial role in the acute phase response to inflammation and infection. It is synthesized by various cell types, including macrophages, dendritic cells, fibroblasts, mesenchymal cells, and glial cells, in response to pathogenic stimuli or inflammatory conditions [47] (Table 3). Elevated PTX-3 levels in blood samples have consistently been associated with more severe cases of sepsis and unfavorable outcomes. Therefore, the measurement of PTX-3 levels can assist healthcare professionals in promptly identifying and treating sepsis, offering valuable insights into the severity of the infection and the patient’s response to therapy [91].

While PTX-3 is not yet included in the major sepsis guidelines, such as the SSC guidelines, ongoing research supports its potential utility as a sepsis biomarker. Some studies have highlighted the added value of PTX-3 in combination with other biomarkers like procalcitonin in improving the accuracy of sepsis diagnosis and prognosis. In a multicenter trial, the clinical and prognostic value of PTX-3 was investigated in a cohort of 958 patients diagnosed with severe sepsis or septic shock. Plasma PTX-3 levels were measured on days 1, 2, and 7 post-randomization, with patients receiving either albumin or crystalloids for fluid resuscitation. Initial PTX-3 levels were elevated (72 ng/mL) and correlated significantly with the severity of organ dysfunction and the occurrence of new failures (*p* < 0.001). Although PTX-3 levels declined from day 1 to day 7, this reduction was less pronounced in patients with septic shock (*p* = 0.0004). Higher PTX-3 levels on day 1 were predictive of developing new organ dysfunctions, with albumin supplementation significantly associated with reduced PTX-3 levels in patients with septic shock (*p* = 0.005), but not in those without shock. Additionally, a fully adjusted multivariable model identified PTX-3 levels on day 7 as a predictor of 90-day mortality. Patients with smaller in PTX-3 over time had a higher risk of 90-day mortality. Thus, elevated early PTX-3 levels were indicative of subsequent organ failures, and a less significant decrease in PTX-3 levels over time was linked to increased mortality risk in severe sepsis and septic shock [92]. In a prospective observational analysis, the combined measurement of PTX3, IL-6, PCT, and lactate demonstrated excellent predictive performance for 28-day all-cause mortality in patients with sepsis or septic shock, surpassing the SOFA score [93]. A systematic review and meta-analysis of 16 studies involving 3,001 patients demonstrated that elevated PTX-3 levels significantly correlated with the increased severity of sepsis and higher all-cause mortality. Specifically, PTX-3 was found to be higher in patients with more severe sepsis (standard mean difference = 18.5 ng/mL; standard error: 4.5 ng/mL, *p* < 0.0001) and non-survivors (standard mean difference = 40.3 ng/mL; standard error: 6.8 ng/mL, *p* < 0.0001), with an increased risk of mortality by 91% (hazard ratio: 1.91, 95% CI: 1.53 to 2.46, *p* < 0.0001) [94]. In the study of sepsis-induced acute kidney injury (AKI) in critically ill patients, PTX-3 was identified as a key marker of inflammation and tissue damage. Elevated PTX-3 deposits in renal tissues and increased serum levels were observed in a swine model of lipopolysaccharide (LPS)-induced AKI, indicating significant immune activation and organ damage [95].

#### 5.1.2. Adrenomedullin (ADM)

ADM, primarily produced by vascular smooth muscle and endothelial cells, can be synthesized in various tissues, including the adrenal cortex, kidneys, lungs, blood vessels, and heart. ADM displays multiple biological properties, such as vasodilation, positive inotropy, diuresis, natriuresis, and bronchodilation, and it has the ability to reduce endothelial permeability by regulating vascular activity [19].

Research has demonstrated that ADM levels correlate with sepsis severity, organ failure, and 30-day mortality, highlighting its diagnostic and prognostic utility. Elevated ADM levels in sepsis patients are linked to higher mortality, underscoring its clinical importance in managing critically ill patients. In a study involving 215 patients (109 with sepsis and 106 with septic shock), ADM quartiles were associated with the number of organ failures, the SOFA score, and impairments in cardiovascular, renal, coagulation, and liver function. Furthermore, ADM levels were able to accurately predict the 30-day mortality, showing a comparable performance to the SOFA score (AUC: 0.827 vs. 0.830). A retrospective observational trial involving 1867 patients (632 with sepsis and 267 with septic shock) found ADM levels to be 74 pg/mL in sepsis patients, 107 pg/mL in patients with septic shock, and 29 pg/mL in patients without sepsis. Elevated ADM levels correlated with increased mortality in both sepsis and ICU patients, with odds ratios of 1.23 and 1.22, respectively [96].

Studies have shown that the stable mid-regional fragment of proadrenomedullin (MR-proADM), consisting of the 45–92 amino acid sequences of the preproadrenomedullin, possesses a prolonged half-life and can serve as an indirect indicator of ADM levels in the body. It is easily detectable and has been used as a biomarker for sepsis and septic shock. One study identified MR-proADM as a predictor of failure across the following five organ systems: respiratory, coagulation, cardiovascular, neurological, and renal systems [97]. Compared to other biomarkers and clinical scores, MR-proADM more accurately identified patients at risk of disease progression [98]. A study evaluated MR-proADM alongside PCT and copeptin in 90 patients, including 28 with sepsis, 32 with septic shock, and 30 control subjects. The findings revealed that MR-proADM levels were significantly different between the sepsis and septic shock groups, and notably higher in patients who did not survive, indicating its relevance in assessing the severity and prognosis of sepsis. Furthermore, a significant correlation was identified between the MR-proADM levels and the length of hospital stay. These results suggest that MR-proADM can serve as a valuable biomarker for diagnosing sepsis, determining its severity, and predicting patient outcomes, including mortality and hospitalization duration [99]. In a randomized controlled trial involving 1089 patients (142 with sepsis and 947 with septic shock), MR-proADM levels within the initial 24 h post-diagnosis correlated with 7-day mortality (AUC: 0.72, *p* < 0.001) and 90-day mortality (AUC: 0.71, *p* < 0.001). Patients exhibiting consistently elevated MR-proADM levels on days 1 and 4 had significantly higher mortality risks, with odds ratios of 19.1 (8.0–45.9) and 43.1 (10.1–184.0), respectively [100]. Additionally, MR-proADM can potentially be eliminated through continuous renal replacement therapy (CRRT) [101]. While ADM has shown promise as a sepsis biomarker, its incorporation into clinical guidelines or recommendations is still evolving. Some studies have suggested the potential utility of ADM as part of a biomarker panel for sepsis diagnosis and management. However, further research and validation are needed before the widespread adoption of ADM testing in routine clinical practice.

#### 5.1.3. Endothelial Cell-Specific Molecule-1 (ESM-1)

ESM-1 is a soluble proteoglycan secreted by endothelial cells, which is regulated by vascular endothelial growth factor and pro-inflammatory cytokines in sepsis patients and COVID-19 patients [102]. Studies have shown that a serum ESM-1 level exceeding 6.28 ng/mL can serve as a prognostic indicator for mortality in individuals with sepsis, exhibiting a sensitivity of 75.9% and specificity of 61.3%. Furthermore, each incremental increase of 1 ng/mL in serum ESM-1 levels is associated with an 11.1% rise in the risk of mortality [103].

In a study involving 56 COVID-19 patients, serum endocan levels were notably higher compared to the controls, indicating its potential as a biomarker for endothelial dysfunction and inflammation. However, the study found no significant correlation between endocan levels and COVID-19 severity or the presence of cardiovascular diseases, suggesting its primary utility may be in the initial detection of inflammatory states rather than as a predictor of disease severity [104]. In addition, in a study investigating the efficacy of various biomarkers for diagnosing late-onset neonatal sepsis, ESM-1 did not show significant differences in serum concentrations among groups, which included symptomatic and infected newborns, symptomatic but uninfected, and asymptomatic controls. Unlike PCT and IL-6, which demonstrated high sensitivity and predictive values, ESM-1’s performance suggests that it may not be a reliable biomarker for this condition [105]. ESM-1 may not be included in current guidelines or recommendations for sepsis management, as it is still being evaluated in ongoing clinical studies. It is essential for healthcare providers to stay updated on the latest research findings and guidelines related to sepsis biomarkers to incorporate them effectively into clinical practice.

#### 5.1.4. Plasminogen Activator Inhibitor-1 (PAI-1)

PAI-1 serves as an indicator for abnormal fibrinolysis in patients. Having significance as a biomarker in coagulation, PAI-1 has been extensively examined in sepsis [106]. Studies have shown that PAI-1 levels can predict the 28-day mortality rate in sepsis patients, with a critical concentration threshold of 83 ng/mL [107]. When the PAI-1 concentration exceeds 83 ng/mL, patients are at a higher risk of developing disseminated intravascular coagulation (DIC) alongside multiple organ dysfunction syndrome (MODS).

Research on 181 septic patients showed that high serum levels of PAI-1, particularly in the presence of the rs1799768 SNP (4G/4G and 4G/5G), were significantly associated with increased 28-day mortality (odds ratio [OR] 3.36; 95% CI 1.51, 7.49). These findings suggest that PAI-1 could serve as a biomarker for prognosis in sepsis, influencing clinical outcomes by regulating neutrophil activity and inflammatory responses [108]. Elevated plasma levels of PAI-1 correlate positively with disease severity and mortality in sepsis, as well as with acute kidney injury (AKI) in septic patients. A study using a murine model of sepsis showed that old-age PAI-1 knockout mice were significantly more susceptible to sepsis-induced mortality compared to wild-type mice (24% vs. 65% survival, *p* = 0.0037), indicating that PAI-1 plays a protective role in managing sepsis, especially in older individuals. These findings suggest that PAI-1 could be an important biomarker and therapeutic target in sepsis, particularly in mitigating age-related vulnerabilities to the condition [109]. PAI-1 4G/5G polymorphisms have been studied for their potential role in pediatric sepsis, with a meta-analysis revealing an association between these polymorphisms and an increased risk of sepsis in children. The meta-analysis involved twelve case-control studies, covering a total of 860 cases and 1144 controls, and found significant associations, particularly among Caucasian children. These findings suggest that PAI-1 4G/5G polymorphisms could serve as genetic biomarkers for the predisposition to pediatric sepsis, indicating their importance in the prognosis and understanding of the disease [110]. Although PAI-1 is not currently included in major sepsis guidelines or recommendations as a routine biomarker, its potential as a prognostic indicator in sepsis is being increasingly recognized. Research is ongoing to further elucidate the role of PAI-1 in sepsis pathophysiology and its utility as a biomarker for predicting outcomes and guiding treatment strategies.

#### 5.1.5. S100 Calcium-Binding Protein B (S100B)

S100B is a marker of blood–brain barrier disruption, as well as glial cell damage and activation. It is commonly used for assessing the severity of brain injuries and predicting outcomes in conditions such as stroke, traumatic brain injury, encephalopathy, and delirium [111]. A prospective cohort trial revealed that S100B levels measured on the third day were more accurate in predicting 180-day mortality compared to levels measured on the first day (AUC: 0.731 vs. 0.611). In an observational study of 22 patients with septic shock, 10 of whom experienced delirium, an S100B level exceeding 0.15 μg/L was associated with an odds ratio of 18.0 for delirium. Additionally, patients with delirium had higher plasma IL-6 concentrations, and there was a positive correlation between S100B and IL-6 levels [112].

In the context of sepsis-associated encephalopathy (SAE), which frequently affects older individuals and primarily presents as delirium without focal neurological symptoms, the S100B protein emerges as a promising biomarker for quantifying neuronal and axonal injury. Elevated S100B levels have also been observed in patients with sepsis-associated encephalopathy (SAE) [113]. A systematic review and meta-analysis of 28 studies involving 1401 SAE patients and 1591 non-encephalopathy septic patients was performed. The findings revealed that SAE patients exhibited significantly higher serum S100B levels compared to the controls (*p* < 0.00001), and these levels were notably elevated in septic patients with burns (*p* < 0.0002). Furthermore, septic patients with favorable outcomes had significantly lower serum S100B levels than those with unfavorable outcomes (*p* < 0.00001), highlighting its diagnostic and prognostic relevance in SAE [114]. While routine imaging and laboratory tests such as CRP and procalcitonin monitor sepsis, they fall short in specifically identifying septic encephalopathy. Elevated S100B levels, along with neurofilament light chains (NfL), have been identified as key indicators for predicting delirium and unfavorable outcomes in patients suffering from septic encephalopathy, underscoring their potential as specific and prognostic biomarkers for this condition [115]. As for clinical use, S100B may provide valuable information in combination with other biomarkers and clinical parameters for assessing the severity of sepsis and predicting outcomes. Currently, recommendations or guidelines regarding the use of S100B specifically as a sepsis biomarker may be limited. As research in this area continues to evolve, it is essential to stay updated on the latest findings and guidelines from relevant medical societies or organizations.

#### 5.1.6. N-Terminal-Pro Hormone BNP (NT-proBNP)

NT-proBNP is an inactive prohormone produced by the heart in response to elevated cardiac pressure. Elevated levels of NT-proBNP within the first 24 h of sepsis onset are linked to lower Short Physical Performance Battery scores at 12 months and reduced grip strength at both 6 and 12 months post-sepsis.

Thus, NT-proBNP levels during the acute phase of sepsis could serve as valuable predictors for the long-term risk of impaired physical function and muscle strength in sepsis survivors [116]. Increased NT-proBNP in sepsis patients reflects cardiac stress and potential dysfunction, which may complicate the clinical course of sepsis. Monitoring NT-proBNP levels can provide critical insights into the cardiovascular status of sepsis patients, aiding in prognosis and potentially guiding therapeutic interventions to improve patient outcomes. Another study found that non-survivors had significantly higher NT-proBNP levels upon admission compared to survivors (7908 vs. 3479 pg/mL). The AUC values for NT-proBNP levels at admission and 72 h post-admission in predicting in-hospital mortality were 0.631 and 0.648, respectively [117]. In patients with sepsis, elevated levels of NT-proBNP have been identified as significant predictors of left ventricular systolic dysfunction (LVSD), a condition linked with higher mortality and complications like atrial fibrillation. The study established a cutoff value for NT-proBNP at ≥3270 pg/mL as part of a predictive model that effectively identified sepsis patients at risk for LVSD. NT-proBNP, alongside other markers such as high-sensitive troponin I (Hs-TnI), PCT, and lactate, proved to be reliable in diagnosing cardiac dysfunction in sepsis [118]. In a prospective cohort study, NT-proBNP was evaluated for its effectiveness in predicting in-hospital sepsis-related mortality among patients admitted to emergency departments. While other assessment scores like the National Early Warning Score 2 and qSOFA were more effective in detecting initial sepsis and septic shock, NT-proBNP demonstrated superior performance in estimating the risk of sepsis-related mortality. The integration of NT-proBNP measurements into prehospital assessments could significantly enhance the prognostic accuracy for sepsis-related outcomes [119]. Although the use of NT-proBNP as a sepsis biomarker is still evolving, some guidelines and recommendations have started to incorporate its use in the management of septic patients. For instance, the SSC guidelines mention the potential role of NT-proBNP in assessing fluid responsiveness and guiding fluid therapy in septic patients with signs of cardiac dysfunction.

#### 5.1.7. Non-Coding RNAs

Long non-coding RNAs play crucial roles in processes such as growth, development, cell proliferation, differentiation, and apoptosis. Studies have shown that the expression of the lncRNA CASC2 is reduced in sepsis patients and inversely correlated with APACHE II and SOFA scores, as well as TNF-α, IL-1β, and IL-17A levels. Furthermore, lncRNA CASC2 levels are lower in sepsis non-survivors compared to survivors, suggesting its potential in predicting the 28-day mortality among sepsis patients [120].

High levels of microRNA-155 expression have been linked to a poorer prognosis in sepsis patients [121]. In sepsis patients, lncRNA metastasis-associated lung adenocarcinoma transcript 1 (lnc-MALAT1) and miR-125a are positively correlated with APACHE-II and SOFA scores, making the lnc-MALAT1/miR-125a axis a predictor of increased 28-day mortality risk [122]. Furthermore, the ratio of lncRNA maternally expressed gene 3 (lnc-MEG3) to lnc-MEG3/miR-21 serves as a robust indicator of increased sepsis vulnerability, whereas miR-21 presents as a reliable marker for decreased sepsis susceptibility [123]. Additionally, elevated levels of miR-125a and miR-125b have been observed in sepsis patients and offer predictive value for sepsis risk assessment. Specifically, while miR-125a proves inadequate in forecasting 28-day mortality in sepsis patients (AUC: 0.588), miR-125b demonstrates superior predictive performance (AUC: 0.699) [122]. More validation studies and clinical trials are needed to confirm the reliability and reproducibility of using lncRNA CASC2 as a biomarker for sepsis. As the field of RNA biomarkers continues to evolve, future guidelines and recommendations for sepsis management may consider incorporating lncRNA CASC2 alongside traditional biomarkers.

#### 5.1.8. Others

In a prospective multicenter cohort trial, 483 sepsis patients were monitored for up to a year. The study found that patients exhibiting high inflammation markers (IL-6 and high-sensitivity CRP) and an immune-suppressed profile (elevated sPD-L1 levels) had increased rates of readmission and mortality, including deaths related to cardiovascular and cancer causes [124]. Another study assessed the clinical significance of IL-6, PTX-3, and PCT in patients with sepsis and septic shock. Serum levels of IL-6, PTX-3, and PCT were measured in 142 participants to assess their prognostic value. The findings indicated that the high-IL-6 group had significantly higher 28-day mortality rates compared to the low-IL-6 group, identifying IL-6 as a risk factor for 28-day mortality [47].

A recent prospective observational trial of 114 patients demonstrated that the expression levels of four signaling molecules (PD-1, CD28, PD-L1, and CD86) on NK cells were directly related to SOFA scores. In high-antigen-load conditions like sepsis, the abnormal upregulation of PD-1/PD-L1 can disrupt both innate and adaptive immune systems, leading to multi-organ failure and potentially death. PD-1 is emerging as a novel prognostic biomarker that could improve the predictive accuracy of the SOFA score in sepsis patients. Furthermore, the high expression of CD39+ Tregs in sepsis is positively correlated with SOFA scores and mortality, suggesting a poor prognosis [125]. Persistently low expression of mHLA-DR is linked to poor outcomes in patients with septic shock. A significant decrease in mHLA-DR/CD14+ expression 48 h after the onset of septic shock markedly increases the 28-day mortality rate [126]. Another study showed that CD13, CD64, and HLA-DR exhibit acceptable sensitivity and specificity for predicting mortality (CD13 AUC: 0.824; CD64 AUC: 0.843; HLA-DR AUC: 0.804), whereas CD14 and CD25 do not reliably predict mortality [127]. In a study involving 49 sepsis patients, 34 developed DIC, leading to eight fatalities. Patients with DIC had lower C3 levels and higher SC5b-9 levels. Stratification based on SC5b-9 quartiles (low: ≤260 ng/mL; medium: 260–342 ng/mL; high: 343–501 ng/mL; highest: >501 ng/mL) demonstrated that patients in the highest quartile displayed the most disrupted coagulation parameters, prolonged thrombocytopenia, and higher mortality rates [128].

In a study involving 51 sepsis patients, plasma levels of occludin (OCLN), claudin-5 (CLDN-5), zonula occludens-1 (ZO-1), PCT, and lactate were evaluated. OCLN and ZO-1 levels increased with disease severity and correlated positively with APACHE-II, SOFA scores, and lactate levels. ZO-1 exhibited a predictive accuracy for in-hospital mortality comparable to that of lactate, APACHE-II, and SOFA scores, superior to OCLN and PCT [129]. Another study evaluated soluble FMS-like tyrosine kinase 1 (sFlt-1), soluble E-selectin (sE-selectin), soluble intercellular adhesion molecule 1 (sICAM-1), soluble vascular cell adhesion molecule 1 (sVCAM-1), and PAI-1. All of these endothelial biomarkers were associated with sepsis severity, with sFlt-1 exhibiting the most robust correlation with the SOFA scores, and both sFlt-1 and PAI-1 having the highest AUC values for mortality prediction [130].

### 5.2. Novel Prognostic Biomarkers

#### 5.2.1. Prokineticin 2

Prokineticin 2 was initially identified as a gastrointestinal peptide involved in peristalsis regulation. However, further research has demonstrated that prokineticin 2 is widely expressed in various tissues, including the central nervous system (CNS), non-steroidogenic cells of the testes, and immune cells. As a secretory protein, prokineticin 2 governs multiple biological processes. Studies have indicated a significant decrease in prokineticin 2 levels in patients with sepsis and septic shock compared to healthy individuals, with this decrease strongly correlating with sepsis progression. Moreover, administering recombinant prokineticin 2 to both heterozygous prokineticin 2-deficient mice and wild-type mice prevented sepsis-related mortality and alleviated the multi-organ damage caused by sepsis. These findings suggest that prokineticin 2 could serve as a valuable prognostic biomarker for sepsis, underscoring its crucial role in reducing sepsis-induced mortality and providing a novel approach for sepsis immunotherapy [131]. Although prokineticin 2 has shown promise as a sepsis biomarker in research studies, its incorporation into clinical guidelines or recommendations is still evolving. As more research is conducted and validated, there is the potential for PK2 to be included in future guidelines for the management of sepsis.

#### 5.2.2. Protein C (PC)

PC, the precursor to activated protein C, is a vitamin K-dependent glycoprotein found in plasma [132]. Primarily known for its anticoagulant function, PC modulates the coagulation pathway by inhibiting Factors V and VIII, which in turn reduces fibrin formation and the activation of platelets and other coagulation factors [133]. A meta-analysis of 12 studies assessing the prognostic value of PC showed that PC levels were significantly higher in sepsis survivors compared to non-survivors, and in patients without DIC compared to those with DIC. Nonetheless, this analysis was constrained by a high risk of bias and insufficient data on sensitivity and specificity. Further research is necessary to confirm the clinical utility of PC as an early prognostic biomarker for sepsis [20,134]. In critically ill sepsis patients, molecular phenotypes resembling those found in ARDS, classified as hypoinflammatory and hyperinflammatory, demonstrated significant differences in outcomes and biomarker profiles, including PC levels. Lower PC levels were observed in the hyperinflammatory phenotype, which also exhibited higher mortality rates, increased vasopressor usage, and more prevalent bacteremia, suggesting that PC could serve as a prognostic biomarker for severity in sepsis. The variation in PC levels among different sepsis phenotypes indicates its potential utility in tailoring treatments and predicting patient responses, particularly to therapies like activated PC, where the effectiveness varied significantly based on the phenotype [135]. PC testing is not yet a routine part of sepsis management guidelines, and some studies have highlighted its potential as a valuable biomarker in the diagnosis and prognostication of septic patients. Incorporating PC testing into sepsis management protocols may help in identifying patients at a high risk of complications and guiding the use of targeted therapies to improve outcomes.

**Table 3:** List of prognostic sepsis biomarkers.

Biomarker	Source	Biological Function	Clinical Applications	Testing Methods	Strengths	Limitations	Refs.
Commonly used prognostic biomarkers							
PTX-3	Various cells (macrophages, dendritic cells)	Acute inflammatory response	Early elevation in PTX-3 levels predicts organ failure Decreasing PTX-3 levels over time are associated with an increased risk of mortality	ELISA Luminex Assay Western Blot	High sensitivity for early organ failure prediction	Limited specificity in non-septic inflammatory conditions	[47,91,92,93,94,95]
ADM	Vascular smooth muscle and endothelial cells	Vasodilation, reduced endothelial permeability	Elevated ADM levels are associated with 30-day mortality and can predict organ failure	CLIA ELISA IRMA	Strong association with mortality and organ failure	Variable response in different patient populations	[19,96,97,98,99,100]
ESM-1	Endothelial cells	Regulation of angiogenesis and inflammation	Each 1 ng/mL increase in ESM-1 levels is associated with an 11.1% increase in the risk of mortality ESM-1 threshold for predicting mortality is 6.28 ng/mL	ELISA Western Blot IHC	Predictive value for mortality	Limited clinical studies	[102,103,104,105]
PAI-1	Various cells (endothelial cells, platelets, and adipocytes, etc.)	Inhibition of fibrinolysis	PAI-1 levels > 83 ng/mL are associated with DIC and MODS Elevated PAI-1 levels predict mortality within 28 days	ELISA Luminex Assay Chromogenic Assay	Strong association with DIC and MODS	Variable response in different patient populations	[106,107,108,109,110]
S100B	Glial cells	Reflects blood–brain barrier disruption and brain injury	S100B levels on day 3 are better predictors of 180-day mortality than levels on day 1 Patients with sepsis-associated encephalopathy exhibit heightened S100B levels	ELISA ECLIA IRMA	Strong association with brain injury and mortality	Limited to central nervous system involvement	[111,112,113,114,115]
NT-proBNP	Cardiac ventricular myocytes	Response to cardiac pressure changes	Elevated NT-proBNP levels within 24 h of sepsis onset are associated with long-term physical function and muscle strength impairment Increased NT-proBNP levels upon admission serve as predictors of in-hospital mortality	ECLIA ELISA Point-of-care testing	High sensitivity for cardiac dysfunction	Limited specificity in non-cardiac conditions	[116,117,118,119]
lncRNAs CASC2	Various tissues	Regulation of gene expression, cell proliferation, differentiation, apoptosis	The lncRNA CASC2 levels are lower in sepsis non-survivors and can predict 28-day mortality risk Lower levels of lncRNA CASC2 are associated with higher APACHE II and SOFA scores.	qRT-PCR RNA-FISH RNA-seq	Strong association with mortality risk and clinical scores	Limited clinical validation	[120,121,122]
miRNAs	Various cells	Post-transcriptional regulation of gene expression	High miR-155 expression indicates a poorer prognosis Both miR-125a and miR-125b predict sepsis risk and 28-day mortality	qRT-PCR NGS Microarray Analysis	Strong association with sepsis risk and mortality	Limited clinical studies and validation	[84,90]
sPD-L1	Immune cells and tumor cells	Immune suppression	High sPD-L1 levels are associated with high readmission and mortality rates in sepsis patients sPD-L1 enhances SOFA score’s predictive ability in sepsis patients	ELISA Luminex Assay Western Blot	Strong association with immune suppression and mortality	Limited to specific immune responses	[124,125]
Novel prognostic biomarkers							
Prokineticin 2	Various tissues (CNS, gastrointestinal tract, and immune cells).	Regulation of multiple biological processes	Prokineticin 2 levels in sepsis patients correlate with disease progression Recombinant prokineticin 2 reduces sepsis-related mortality in mice	ELISA RIA Western Blot	Strong association with disease progression	Limited clinical studies and validation in human subjects	[131]
PC	Liver	Anticoagulant, regulates coagulation cascade	High PC levels in sepsis survivors predict early sepsis Meta-analysis shows PC levels higher in survivors	ELISA Chromogenic Assay Clotting Assay	Strong association with survival and early sepsis detection	Limited availability in routine clinical practice	[20,132,133,134,135]

CLIA, chemiluminescence immunoassay; DIC, disseminated intravascular coagulation; ECLIA, electrochemiluminescence immunoassay; ELISA, enzyme-linked immunosorbent assay; IRMA, immunoradiometric assay; MODS, multiple organ dysfunction syndrome; NGS, next-generation sequencing; RIA, radioimmunoassay; qRT-PCR, quantitative real-time PCR; RNA-FISH, RNA fluorescence in situ hybridization; RNA-seq, RNA sequencing; SOFA, sequential organ failure assessment.

## 6. Biomarkers for Sepsis-Associated Acute Kidney Injury (Sepsis-AKI)

Sepsis-AKI is a common and severe complication in critically ill patients, significantly increasing morbidity and mortality [136,137]. Currently, no approved pharmacological therapies exist to either prevent sepsis-AKI or to treat sepsis-AKI once it occurs [138]. Sepsis-AKI is a complex clinical syndrome that involves multiple pathophysiological mechanisms, including inflammatory responses, hemodynamic alterations, microcirculatory disturbances, and cellular injury [139]. Numerous biomarkers have been studied and identified in the context of sepsis-AKI, each playing a distinct role in pathology.

### 6.1. Inflammatory Biomarkers

Produced by immune cells and renal tubular cells, suPAR is also associated with immune activation and inflammation. It is a predictor of AKI and its progression in septic patients [140]. A 2023 meta-analysis linked high suPAR levels with increased risk of AKI, suggesting its potential as a prognostic biomarker [141].

### 6.2. Microcirculatory Disturbance Biomarkers

Microvascular endothelial cells in the kidney have been a neglected cell type in sepsis-AKI research; yet, they offer tremendous potential as pharmacological targets [142]. Two clinical studies have shown that plasma-free hemoglobin (CFH) levels are significantly elevated in some patients with sepsis, and that patients with higher CFH levels have an increased risk of death and septic acute kidney injury [143,144]. Bedside measurements of CFH levels may help predict clinical trials of CFH-targeted therapies in order to recruit patients with elevated CFH who are most likely to benefit. However, in order to advance such trials, rapid and accurate bedside tests of plasma CFH need to be developed [138].

Vascular Endothelial Growth Factor (VEGF) is produced by various cells, including renal tubular cells, in response to hypoxia and inflammation. VEGF plays a role in angiogenesis and vascular permeability. In sepsis-AKI, altered VEGF levels are associated with endothelial dysfunction and increased vascular permeability, contributing to kidney injury. VEGF levels can be used as a marker of endothelial damage and inflammation in sepsis-AKI. Monitoring VEGF levels may help assess the severity of kidney injury and the effectiveness of therapeutic interventions. The role of VEGF in sepsis-AKI is complex, as both high and low levels have been associated with different outcomes. This dual role may complicate its use as a straightforward biomarker [142].

ESM-1, also known as endocan, is a proteoglycan produced by endothelial cells. ESM-1 is involved in the regulation of vascular permeability and inflammation. Elevated ESM-1 levels are associated with endothelial dysfunction and have been linked to the severity of sepsis-AKI. ESM-1 serves as a marker for endothelial activation and inflammation. Its levels can predict the risk of developing AKI in septic patients and may be used to monitor disease progression. The utility of ESM-1 as a biomarker may be limited by its variability in different clinical conditions and patient populations, necessitating further validation in large-scale studies [145].

## 7. Multi-Biomarker Approach

The utilization of a multi-biomarker approach in sepsis management and diagnosis has gained significant attention due to its potential to enhance the accuracy of identifying and assessing sepsis. Sepsis is a multifaceted condition with diverse pathophysiological mechanisms, and relying on a single biomarker may not capture the full spectrum of the disease. By integrating multiple biomarkers that reflect different aspects of the immune response, inflammation, and organ dysfunction, a more comprehensive evaluation of the patient’s condition can be achieved. For instance, PCT indicates bacterial infection, while elevated lactate levels indicate tissue hypoperfusion and organ dysfunction. A combination of these markers can provide insights into both the infectious and metabolic aspects of sepsis. CRP serves as a general marker of inflammation, while IL-10 and TNF-α are specific cytokines associated with the immune response. A combination of these biomarkers can offer a comprehensive evaluation of the inflammatory cascade in sepsis. Moreover, suPAR serves as a marker of immune activation and prognosis in sepsis, while ESM-1 reflects endothelial dysfunction. Integrating these markers can provide insights into both the immune response and vascular involvement in sepsis.

The multi-biomarker approach plays a crucial role in the early detection of sepsis and the stratification of patients based on their risk of progression to severe sepsis or septic shock. By incorporating multiple biomarkers, clinicians can customize treatment plans based on the individual patient’s response to therapy and disease progression. The combination of biomarkers allows for a more holistic assessment of the pathophysiology of sepsis, leading to a more accurate diagnosis and monitoring of the disease. Despite its advantage, the multi-biomarker approach is not without limitations. Biomarker levels can vary widely among individuals and may be influenced by factors such as age, comorbidities, and medications. Standardizing the interpretation of multi-biomarker panels poses a challenge. Some biomarkers may be costly to measure, and their availability in clinical settings may be limited, particularly in resource-constrained environments. Integrating the results of multiple biomarkers requires expertise and may pose challenges in clinical decision making, especially when faced with conflicting or inconclusive results.

## 8. Conclusions

**Figure 1:**
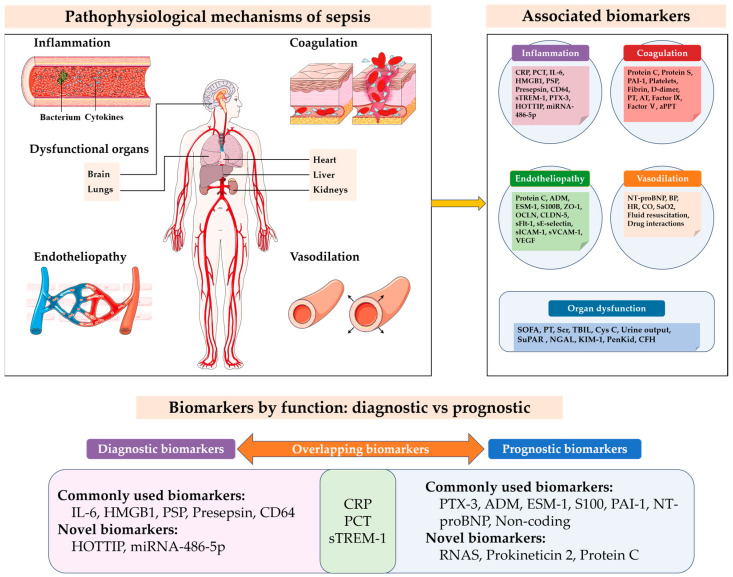
Pathophysiological mechanisms and associated biomarkers of sepsis. The biomarkers are categorized based on their diagnostic and prognostic roles, with a distinction made between traditional and novel biomarkers. Some biomarkers are shown to have dual functions, serving both diagnostic and prognostic purposes, reflecting their overlapping roles in sepsis management. The distribution of biomarkers in the figure is designed to represent their relevance of use in clinical practice. The clipart used in this figure has been sourced and adapted from Servier’s Medical Art database (https://smart.servier.com (accessed on 20 June 2024).

Early diagnosis and prognosis play a critical role in enhancing patient outcomes in sepsis. This review has delved into various biomarkers, each providing valuable insights into the pathophysiology, severity, and prognostic prospects of sepsis. These biomarkers hold promise in enabling early diagnosis, monitoring disease progression, and predicting outcomes. For a comprehensive understanding of the links between the biological processes and biomarkers investigated in sepsis, please refer to Figure 1. This diagram concisely illustrates the vital pathways of inflammation, coagulation, endothelial dynamics, and vasodilation along with their corresponding biomarkers, showcasing the intricate nature of sepsis. The development of cost-effective, non-invasive, and rapid testing methods for these biomarkers will be pivotal for their integration into routine clinical practice; however, challenges such as variability in biomarker levels among diverse patient populations and clinical settings need to be tackled. Given the complexity of sepsis, a multi-biomarker approach is essential to enhance the diagnostic and prognostic precision. Combining various biomarkers can improve the sensitivity and specificity by capturing different aspects of the host response to infection. Further exploration into multi-biomarker panels may offer a more holistic assessment of sepsis, facilitating tailored treatment strategies and better patient outcomes. Moreover, future research should center on conducting large-scale, multicenter trials to validate the efficacy and reliability of these biomarkers. The exploration of combined multiple biomarkers could elevate the diagnostic accuracy and prognostic precision. Technological advancements and bioinformatics could pave the way for novel biomarkers with enhanced specificity and sensitivity. In conclusion, while no single biomarker can fully encapsulate the complexity of sepsis, the combined utilization of multiple biomarkers displays potential in advancing sepsis management. Ongoing research and innovation are crucial for surpassing current limitations and achieving more effective, personalized approaches to sepsis diagnosis and treatment.

## Figures and Tables

**Table 1:** Published reviews of sepsis biomarkers.

No.	Title (Year of Publication)	Key Points	Refs.
1	Biomarkers for the prediction and judgement of sepsis and sepsis complications: a step towards precision medicine? (2022)	A total of 17 biomarkers aid in assessing the inflammatory status and guiding immunomodulatory therapy for sepsis-related systemic inflammation. Biomarkers have been exemplified in guiding the treatment for SIRS, specific therapy (e.g., antibody therapy), and managing complications like acute kidney injury. Case studies show how biomarkers improve clinical management in complex conditions like septic shock, including sepsis-associated acute kidney injury. Future biomarker studies can help select more homogeneous cohorts, improving research conditions for clinical trials and exploring omics technologies’ prospects.	[11]
2	Biomarkers of sepsis: time for a reappraisal (2020)	A review spanning from 2009 to 2019 identified 258 sepsis biomarkers, with over 80 new additions. Only a small percentage of biomarkers underwent robust evaluation, with 31% evaluated in just one study. Limited progress has been observed in identifying clinically significant biomarkers for sepsis.	[15]
3	Biomarkers predicting tissue pharmacokinetics of antimicrobials in sepsis: a review (2022)	Biomarkers predicting antibiotic target concentrations offer a potential therapeutic avenue for sepsis treatment. Identification of 59 biomarkers capable of guiding targeted antibiotic dosing in critically ill patients, considering various factors such as host factors and patient pharmacokinetic variations. Limited evidence exists regarding the clinical significance of many biomarkers, yet proposed biomarkers show promise for optimizing ICU antibiotic therapy.	[16]
4	How to use biomarkers of infection or sepsis at the bedside: guide to clinicians (2023)	Introduction of 11 pathogen-specific biomarkers and commonly used host-response biomarkers like PCT and CRP to enhance sepsis patient care. Review of the roles of pathogen-specific and host-response biomarkers and their clinical evidence in improving sepsis patient management. Emphasis on the need for large multicenter cohort studies utilizing advanced technologies like omics, bioinformatics, and machine learning to identify biomarkers predicting responses to specific interventions.	[17]
5	Current evidence and limitation of biomarkers for detecting sepsis and systemic infection (2020)	Introduction of 6 promising sepsis biomarkers (CRP, PCT, IL-6, CD64, procalcitonin, and sTREM-1) and their clinical evidence. Recognition of CD64 and presepsin as the most promising biomarkers for sepsis diagnosis. Recommendations for future studies to utilize larger sample sizes in cohort designs rather than case-control studies to improve biomarker research. Evaluation of research limitations, including sampling strategies, overestimation of biomarker effects, and heterogeneity in study design and analysis methods.	[18]
6	An update on sepsis biomarkers (2020)	Emphasis on identifying patients at risk of sepsis before organ dysfunction occurs, highlighting 17 biomarkers for predicting sepsis diagnosis, prognosis, and treatment response. Classification of newly discovered biomarkers into diagnostic and prognostic categories, with emphasis on their roles in predicting sepsis diagnosis, prognosis, and treatment response. Overview of novel biomarkers, including miRNAs, lncRNAs, and the human microbiome, for their potential in sepsis management. Future clinical applications necessitate further assessment of new biomarkers’ roles in sepsis pathogenesis and the development of standardized analysis strategies.	[19]
7	Biomarkers for sepsis: more than just fever and leukocytosis—a narrative review (2022)	Assessment of whether biomarkers in sepsis patients or those with septic shock can predict mortality, MODS, or organ dysfunction. Discussion on 51 categories of sepsis biomarkers, including fluid-phase PRMs, complement system components, cytokines, chemokines, DAMPs, ncRNAs, miRNAs, cell membrane receptors, cell proteins, and metabolites, highlighting their roles in predicting mortality, MODS, etc. Emphasis on the need for extensive research to identify optimal combinations of biomarkers to improve diagnosis, treatment, and patient outcomes.	[20]

CD64, cluster of differentiation 64; CRP, C-reactive protein; DAMPs, damage-associated molecular patterns; ICU, intensive care unit; IL-6, interleukin-6; strem-1, soluble triggering receptor expressed on myeloid cells-1; miRNAs, microRNAs; MODS, multiple organ dysfunction syndrome; ncRNAs, non-coding RNAs; PCT, procalcitonin; PRMs, pattern recognition molecules; SIRS, systemic inflammatory response syndrome.

**Table 2:** List of diagnostic sepsis biomarkers.

Biomarker	Source	Response Time	Diagnostic Accuracy	Clinical Significance	Testing Methods	Strengths	Limitations	Refs.
Commonly used diagnostic biomarkers								
CRP	Liver	Rises within 4–6 h after infection	AUC: 0.76, 95% CI [0.68–0.84] Sensitivity: 74.4% Specificity: 65.4% (for Gram-negative sepsis)	Early diagnosis of sepsis Monitoring of post-surgery recovery Combination with PCT for better accuracy Nonspecific for inflammation	ITA ELISA hs-CRP Nephelometry	High sensitivity for inflammation Rapid response	Limited specificity False positives in non-infectious inflammation False negatives in localized infections	[15,22,25,26,27,28,29,30,31,32,33,34]
PCT	Thyroid C cells	Rises within 2–4 h after infection	AUC: 0.72 Sensitivity: 73%,95% CI [59–87%] Specificity: 77%, 95% CI [66–88%]	Early diagnosis of sepsis Optimize antibiotic treatment decisions Prediction of positive blood cultures	CLEIA EIA FIA ELISA Point-of-care testing	Good specificity for bacterial infections Rapid rise after infection	Moderate sensitivity False positives in non-bacterial inflammation Expensive test	[17,27,35,36,37,38,39,40,41,42,43,44,45]
IL-6	Immune and non-immune cells	Peaks within 2 h after infection	AUC: 0.71, 95% CI [0.66–0.76] Sensitivity: 68% Specificity: 83%	Early diagnosis of bacterial sepsis Differentiation between sepsis and septic shock	CLEIA ELISA EIA ECLIA	Rapid response Useful in differentiating sepsis severity	Low sensitivity in some populations Variable levels in non-septic inflammatory conditions	[18,46,47,48]
HMGB1	Immune cells (macrophages, monocytes, and neutrophils)	Increases within 4–8 h after infection	AUC: 0.58; 95% CI [0.35–0.78] Sensitivity: 100% Specificity: 83%	Late mediator of sepsis	Western blot ELISA IHC qRT-PCR	High sensitivity and specificity in experimental settings	Less studied in clinical settings Late response in sepsis progression	[49,50,51,52,53,54,55,56]
PSP	Pancreatic acinar cells	Response time is not well-defined, but it rises rapidly after infection	AUC: 0.75 Sensitivity: 77–86% Specificity: 73–78%	Early diagnosis of sepsis Continuous measurement of hospital-acquired sepsis	ELISA TR-IFMA LFA	Rapid rise after infection Useful for hospital-acquired sepsis monitoring	Limited clinical studies Variable response time	[57,58,59,60,61,62,63]
Presepsin	Macrophages and monocyte cells	Rises within 2 h after infection	AUC: 0.78~0.88 Sensitivity: 70–88% Specificity: 64–81%	Early diagnosis of bacterial sepsis Optimize antibiotic treatment decisions	CLEIA Automated platforms	Rapid response Specific association with Gram-negative sepsis	Expensive test Limited availability in some regions	[64,65,66,67]
CD64	Immune cells (especially neutrophils, monocytes/macrophages)	Upregulated within 6–8 h after infection	AUC: 0.94, 95% CI [0.91–0.96] Sensitivity: 88%, 95% CI [81–92%] Specificity: 88%, 95% CI [83–91%]	Early diagnosis of sepsis in ED and ICU	Flow cytometry FIA	High sensitivity and specificity Widely studied	Expensive test Limited use in routine clinical practice	[68,69,70,71,72]
sTREM-1	Myeloid cells	Elevates within 2–4 h after infection	AUC: 0.72~0.89 Sensitivity: 80–85%; 95% CI [66–91%] Specificity: 75–81%; 95% CI [69–86%]	Early diagnosis of sepsis	ELISA Western blot Multiplex immunoassay	Good sensitivity and specificity Useful for early diagnosis	Limited studies on long-term outcomes Variable levels in different patient populations	[73,74]
Novel diagnostic biomarkers								
circRNAs	Various tissues and cells, especially cancer cells and neural cells	Response time varies depending on the particular circRNA	AUC: 0.78, 95% CI [0.63–0.92] Sensitivity: 55–59% Specificity: 90~95%	Early diagnosis of sepsis Potential molecular therapeutic targets of sepsis Higher specificity than CRP and PCT	qRT-PCR RNA-seq Northern Blotting	High specificity for sepsis Potential for targeted therapies	Limited clinical validation Response time variability	[75,76,77,78,79]
HOTTIP	Embryonic stem cells and various cancer cells	Response time is not well-defined	AUC: 0.847 for ARDS in sepsis, 95% CI [0.78–0.92] Sensitivity: 70~80% Specificity: 60~75%	Early diagnosis of sepsis with ARDS Higher AUC than CRP and PCT	qRT-PCR RNA-FISH RNA-seq	High specificity for ARDS in sepsis Potential therapeutic target	Limited clinical studies Variable response time	[81,82,83]
microRNA-486-5p	Various tissues, particularly in skeletal muscles, lung tissues, and various cancer cells	Response time is not well-defined, but changes within several hours after infection	AUC: 0.914 (sepsis patients vs. healthy subjects) AUC: 0.814 (sepsis patients vs. pneumonia patients) Sensitivity: 72~88% Specificity: 84~92%	Early diagnosis of sepsis Distinguishing sepsis patients from pneumonia patients Higher specificity than CRP and PCT	qRT-PCR NGS Northern Blotting	High specificity for sepsis Differentiates sepsis from other infections	Limited clinical studies Variable response time	[84,85,86,87,88,89,90]

ARDS, acute respiratory distress syndrome; AUC, area under the curve; CLEIA, chemiluminescent enzyme immunoassay; ED, emergency department; EIA, enzyme immunoassay; ELISA, enzyme-linked fluorescent assay; FIA, fluorescent immunoassay; hs-CRP, high-sensitivity CRP; ICU, intensive care unit; IHC, immunohistochemistry; ITA, immunoturbidimetric assay; qRT-PCR, quantitative real-time PCR.
